# Impact of Human–Animal Interactions on Psychological and Physiological Factors Associated With Veterinary School Students and Donkeys

**DOI:** 10.3389/fvets.2021.701302

**Published:** 2021-08-23

**Authors:** Elpida Artemiou, Pippa Hutchison, Marcus Machado, Daria Ellis, Jennifer Bradtke, Mary Mauldin Pereira, Julia Carter, Don Bergfelt

**Affiliations:** ^1^Department of Clinical Sciences, Ross University School of Veterinary Medicine, Basseterre, Saint Kitts and Nevis; ^2^Positive Imprint, Kilcreggan, United Kingdom; ^3^Research Laboratory, Ross University School of Veterinary Medicine, Basseterre, Saint Kitts and Nevis; ^4^Ross University School of Medicine, Miramar, FL, United States; ^5^Counseling Center, Ross University School of Veterinary Medicine, Basseterre, Saint Kitts and Nevis; ^6^Ross University School of Veterinary Medicine, Basseterre, Saint Kitts and Nevis; ^7^Department of Biomedical Sciences, Ross University School of Veterinary Medicine, Basseterre, Saint Kitts and Nevis

**Keywords:** human–animal interactions, veterinary student wellbeing, stress, saliva-cortisol, donkey saliva cortisol

## Abstract

There has been an increased interest in evaluating human–animal interactions and assessing the mutual health and wellbeing. In this study, first-year female and male veterinary school students not paired (*n* = 58) or paired (*n* = 25) with immature (≤9 mo) donkeys (*n* = 13) were engaged in three different types of interactions (1st, hands-off remote learning, 2nd, hands-on passive learning, and 3rd, hands-on active learning) for 30 min each during Week 2 (Time 1), Weeks 5–8 (Time 2), and Week 12 (Time 3) over three, 15-week periods. Student psychological data involved the Penn State Worry Questionnaire (PSWQ) scores collected from the interactive (student-donkey pairs) and non-interactive (no student-donkey pairs) groups and modified Comfort from Companion Animals Scale (CCAS) scores collected from the interactive group during Times 1, 2, and 3. Donkey physiological data involved collection of saliva within 10 min pre- and post-interaction during Times 1, 2, and 3 in association with the different types of interactions for immunoanalysis of cortisol. There were no significant effects of the various times and types of interactions on CCAS scores. While there were no significant effects of group and types of interactions on PSWQ scores, there was an effect (*P* = 0.01) of time. Overall mean PSWQ scores were significantly lower during Week 12 versus Week 2. Correspondingly, while there were no effects pre- vs. post-interaction within or among times on saliva cortisol concentrations in donkeys, there was an effect (*P* = 0.02) of the type of interaction. Mean concentrations were significantly lower with the hands-on passive and hands-on active learning versus the hands-off remote learning. In conclusion, while this study provides preliminary evidence surrounding student donkey interactions, future studies are required with more comprehensive designs to clarify these benefits and better understand the advantages and challenges surrounding student-donkey interactions.

## Introduction

Evidence specific to veterinary medicine identifies increased mental health concerns among veterinary students (1–5). Veterinary students report high levels of academic stress, financial and relational struggles resulting in increased levels of depression, anxiety, suicidal thoughts, problems with substance-abuse, and harmful drinking behaviors (1–5). In response, veterinary institutions continue to explore and develop programs that enhance student wellbeing while collectively acknowledging veterinary students' love for animals and the importance of fostering meaningful social relationships, developing a sense of belonging, engagement, and fulfillment in daily academic student life (6–9).

The Human-Animal Bond (HAB) as defined by the American Veterinary Medical Association (AVMA) is a “mutually beneficial and dynamic relationship between people and animals that is influenced by behaviors that are essential to the health and wellbeing of both” (AVMA, 1998) (10). A review of the benefits of Human-Animal Interactions (HAI) have shown improved social behaviors, reduction of stress based on improved mental and physical health, an overall lowering of fear and anxiety and reduced heart rate, blood pressure, and levels of cortisol (11–13). Extensive evidence exists on the use of horses in animal therapies and studies on Equine-Assisted Activities (EAA) and Equine-Assisted Therapies (EAT). Results indicated improved wellbeing of adolescents in lowering salivary cortisol levels and demonstrated benefits for children with cerebral palsy, autism spectrum disorders, children and adolescents with social, emotional and behavioral difficulties, patients with special needs, psychiatric and substance abuse (14–19). Despite apparent morphological and physiological similarities between horses and donkeys, only recently have donkeys been considered as companion animals used in human-animal therapy (20, 21). Specifically, human-donkey interactions have shown particular benefits surrounding intellectual disability and mental health including managing emotions and improving motivation and communication (20, 21).

In regard to the use of Animal-Assisted Therapies (AAT) with university veterinary students and university owned donkeys, little is known whether the student-donkey interactions can provide social support and an avenue for initiating new social relationships as well as potentially reducing stress and anxiety in students (22–24). In addition, student-donkey interactions may support animal welfare and husbandry practices while extending the role of donkeys as pets and companion animals beyond general use in agriculture, recreation, and teaching and learning in veterinary programs (25–28).

While donkeys can be found on many veterinary school campuses serving as a relevant equine teaching model, many challenges exist when acquiring donkeys for use in learning. During the acquisition process of domesticated or free-ranging donkeys, transportation, and introduction to new herd mates, environmental and social structural changes can potentially impact their health and wellbeing (29, 30).

Human-animal interactions and the relationships that develops between them have been regarded as mutually beneficial and can be comparable to psychological and physiological observations between a parent and child (31) and dog owners and their pets (31–33). Apart from the psychological aspects associated with humans, the physiological and endocrinological aspects associated with both humans and animals have often involved changes in cortisol, body temperature, pulse and respiration, and heart rate (21). In regard to cortisol, domestic animals react to stress through physiological responses, which are the result of individual emotional reactivity (34). Activation of the hypothalamic-pituitary-adrenal (HPA) axis is a prominent neuroendocrine response to emotional or stress-related activities resulting in the increase in systemic concentrations of cortisol in humans (35, 36) and animals (32). While there is no apparent documentation on the impact of student-donkey interactions on cortisol, there are multiple studies that have documented interactions between dog owners and their pets on cortisol and other physiological characteristics (31, 33, 37). Overall, results have indicated decreased cortisol and blood pressure in both humans and dogs (38–42).

The present study is proposed as a preliminary attempt to broaden the scope of human–animal interactions by observing the impact of the interactions between veterinary school students and donkeys on student psychology associated with worry and benefits of an animal companion and donkey physiology associated with temporal changes in saliva cortisol during student-donkey interactions.

## Materials and Methods

### Study Design

The study was conducted from January to December and involved a total of 83 incoming first-year students at Ross Universtiy School of Veterinary Medicine (RUSVM) over 3, 15-week periods or semesters with female and male students ranging from 71–85.5% and 12–14.5%, respectively. Criteria for selection of students included lack of pet ownership while attending RUSVM and non-involvement in research or student clubs that engaged students in continuous animal interactions including fostering and adoption of pets through the duration of the study. Once selected, students had an orientation session outlining the purpose of the study surrounding student-donkey companionship, its potential therapeutic effects, and was approved by the Institutional Animal Care and Use Committee (IACUC) as well as RUSVM safety rules and regulations.

There were 18 adult male and female donkeys from the RUSVM teaching herd. Of the 18 donkeys, there were 13 immature donkeys ≤9 months that were born on RUSVM campus and 5 adult donkeys 12–15 years that were free-ranging prior to capture and transport to RUSVM campus. Females were intact whereas males were castrated in accord with university management practices. The five adult donkeys had been retired from participating in the clinical teaching program, whereas the 13 immature donkeys had not yet been introduced to the veterinary curriculum. During that time, both the mature and immature donkeys had limited interactions with humans. All 18 donkeys were maintained in paddocks with free access to hay, water, and shelter and managed in accordance with RUSVM Animal Husbandry Policy and Procedures as approved by university IACUC.

Male and female students were randomly assigned to an interaction or no interaction (control) groups. There were a total of 25 students in the interaction group and 58 students in the no interaction group. Only students in the interaction group were randomly paired with donkeys by the Animal Resource Manager.

The study encompassed three, 15-week periods over three semesters with new first-year student-donkey pairings each semester. Within each period, student-donkey pairs conducted a 30 min session beginning in Week 2 (Time 1), continuing through Weeks 5 to 8 (Time 2), and ending in Week 12 (Time 3) involving different types of student-donkey interactions. The three levels of student-donkey interactions were conducted sequentially: (1) hands-off remote learning; (2) hands-on passive learning; and (3) hands-on active learning. Students in the control group were not paired with donkeys and, therefore, there were no student-donkey interactions. Student psychological data were collected at Times 1, 2, and 3 for each student-donkey interactive session and donkey physiological data were collected pre- and post-interaction encompassing each interaction level. The study met all IACUC and Institutional Review Board (IRB) RUSVM criteria.

### Student-Donkey Interactions

The student-donkey interactions included three different levels of interactions.

#### Hands Off Remote Learning

Hands off remote learning involved using the paddock door as a natural protective barrier. Students worked with donkeys on touching a target object (long stick with a rubber end) and gave non-verbal cues followed by a reward. The donkey touched the target object as presented from different directions and was rewarded. Once achieved, students could touch the donkey's forehead with a flat hand, cue, and reward. When the student was confident, this work was repeated without a protective barrier. Progressing to the next level required approval by certified veterinary technician who was not directly involved in the study but responsible for animal safety and handling.

#### Hands-On Passive Learning

Hands-on passive learning involved students grooming and leading the donkeys around within the stable, touching and hugging different parts of the donkey, body manipulation such as lifting and stretching front legs, luring head with food as a reward from front to flank and side to encourage full neck stretch and down between front legs.

#### Hands-On Active Learning

Hands-on active learning involved students in external mental stimulation that included working with hand on halter and lead rope and general movement around the paddock. Stepping over two spaced poles on the ground, figure of eight around poles, and moving forward and backward between two poles. Texture challenge was also explored and included—walking over piece of tarpaulin and walking along wide wooden plank (could make soil bank to go up and down on plank). Also included were stepping up/down onto a solid wooden platform and stepping in/out of car-tires placed flat on the ground. Teaching to pick up and hold an object, collect the object (i.e., dumbbell or equi-ball), and target an object at a distance (e.g., a traffic cone).

Within each type of interaction, several stimulating exercises were available to provide choices for each donkey to engage in within a 30-min session. The protocol also offered a degree of flexibility to allow students' variability in comfort levels in interacting with their respective donkeys. All student-donkey interactions were introduced and supervised by student Research Assistants who were not assigned to a donkey and veterinary technical staff who were responsible for safety and animal handling.

### Psychological Assessment of Students

Student psychological data were collected during Time 1 (Week 2), Time 2 (5 to 8), and Time 3 (Week 12), for each of the three, 15-week periods, which included the Penn State Worry Questionnaire (PSWQ) to assess human levels of worry and Comfort from Companion Animals Scale (CCAS) to measure changes associated with Human-Animal Bonding (HAB). The PSWQ is a 16-item, self-report instrument, and is widely used to assess human levels of worry (43, 44). The PSWQ has been shown to identify symptoms of anxiety while not correlating with other symptoms indicative of other psychological disorders and has been used within non-clinical populations with high internal consistency (alpha = 0.83) (43). The instrument rates 16 statements (e.g., If I do not have enough time to do everything I do not worry about it) on five-point Likert type scale from 1 to 5 where 1 = “not at all typical of me” to 5 “very typical of me.”

The CCAS provides a reliable (alpha = 0.85) and quantifiable assessment of HAB (45, 46). The CCAS was initially developed to include items that introduced no significant differences in scores between species (e.g., dogs and cats) (45). However, for the purpose of our study, the scale was modified by changing reference from “pet” to “donkey” such that it evaluated the degree of student attachment to donkeys. CCAS evaluates 13 statements (e.g., “My donkey provides me with companionship”) on a four-point Likert scale where 1 = “strongly disagree” to 4 = “strongly agree.” All human psychological data were interpreted by the Director of the Counseling Center at RUSVM who collaborated in this study.

### Physiological Assessment of Donkeys

Donkey endocrinological data involved collecting saliva samples which is considered a non-invasive approach with minimal to no stress subsequent to conditioning compared to collection of blood samples for assessing cortisol (47, 48). Moreover, salivary cortisol provides direct information about free cortisol concentrations, which is the biologically active fraction. The Salimetrics Enzyme Immune Assay (EIA) kit was validated for use with donkey saliva to quantitate cortisol. Serial dilutions of pools of donkey saliva paralleled the cortisol reference standard curve and spike and recovery of known amounts of low, medium, and high cortisol standards added to donkey saliva resulted in a mean 95% recovery. Thus, results of these assessments for the cortisol EIA provided confidence that we can use these kits to successfully quantify cortisol in saliva samples from donkeys. Comparable validation results of cortisol in donkey saliva have been reported previously (49).

The majority of donkey saliva samples were collected between 0600 and 0900 h. These sampling times involved three student-donkey interaction groups as described (hands-off remote learning, hands-on passive learning, hands-on active learning) with saliva collections within 10 min pre- and post-interaction. Donkey saliva samples were collected using SalivaBio Oral Swab (Salimetrics, State College, PA, USA). For the collection of donkey saliva, the procedure involved the student rinsing the mouth of the animal approximately 5 min before each sampling. The swab was placed on a long stick and inserted in the mouth along the cheek for ~2 min. Thereafter, food was offered as a positive reinforcement which was introduced during a preconditioning period involving 2-3 sessions before the study began. The saliva swab was placed in a collection tube, set on ice, and returned to the laboratory. At the laboratory, donkey and student collection tubes were handled by a laboratory technician who processed the samples. These were then centrifuged at 3,000 rpm for 15 min, removed saliva, stored in labeled cryovials, and froze (−80°C) until hormonal analysis. Concentrations of cortisol in donkey saliva samples were quantitated using a commercial enzyme immunoassay (EIA) kit (Salimetrics, State College, PA, USA) subsequent to validation.

### Statistical Analysis

Data was entered into a Microsoft excel spreadsheet in accordance with guidelines outlined in Broman and Woo (49). Data analysis included descriptive statistics of all variables, including some graphical display of data. All analyses were conducted using SPSS statistical software, version 24 (IBM Corporation, Armonk, New York, USA).

### Student Psychological Data

There were a total of 25 students in the student-donkey interaction group who completed both the PSWQ and the CCAS tests, whereas in the control group there were 58 students who had no interaction with donkeys and completed only the PSWQ test. Only students with scores in all three, 15-week periods were included in the analysis.

Student psychological analyses were conducted separately to test between-subjects and within-subject effects. A two-way mixed ANOVA was applied to test between-subject effects by comparing PSWQ scores between the interaction and control groups across the three, 15-week periods (Times 1, 2, 3). To test for homogeneity of variances, the Mauchly's Test of Sphericity indicated that the assumption of sphericity had not been violated (*P* = 0.178) and the Levene's test of equal variances was not violated (*P* > 0.01).

Similarly, two-way repeated measures ANOVA was applied to test within-subject effects by comparing PSWQ and CCAS scores across the three, 15-week periods. Mauchly's Test of Sphericity indicated that the assumption of sphericity had not been violated (*P* = 0.222) and the Levene's test of equal variances was not violated (*P* > 0.01).

### Donkey Physiological Data

A two-way mixed ANOVA was applied to test within-subject effects by comparing pre- and post-treatment interactions for cortisol concentrations across the three levels of student-donkey interactions. The distributions were positively skewed and the Levene's test of homogeneity of variances were performed indicating that the distributions were not normally distributed. A comparison of the Levene's test of homogeneity of variances for donkey saliva as untransformed and transformed by natural logarithm with a *P* > 0.01 indicates the assumption of homogeneity of variances had not been violated. Outliers were removed using the Tukey's box plot method and all values beyond the threshold were removed and analyses were performed on natural logarithm-transformed pre- and post-cortisol concentrations.

## Results

### Student Psychological Outcomes

There was no main effect of treatment (*P* = 0.28) or interaction of treatment by time (*P* = 0.13). Students in both the control group (36.4 ± 19.8) and interaction group (40.5 ± 16.4) had comparable scores across the three, 15-week periods as shown in Table 1. Overall there was a main effect of time (*P* = 0.01). Mean PSWQ scores were lower (*P* < 0.01) at Time 3 (34.6 ± 20.0) compared to Time 1 (42.0 ± 17.2) (Table 1).

**Table 1:** Combined student PSWQ scores at times 1 (week 2), 2 (weeks 5–8), and 3 (week 12) over three, 15-week periods.

**Groups**	***n***	**TIME 1**	**TIME 2**	**TIME 3**
Control	58	42.1 ± 17.4	34.0 ± 20.0	32.9 ± 20.7
Interaction	25	41.8 ± 17.1	41.2 ± 14.5	38.5 ± 17.9
Total	83	42.0 ± 17.2	36.2 ± 18.8	34.6 ± 20.0^a^

*Student-donkey interactions included 30 min sessions within each of the times periods. Students in the control group were not paired with donkeys*.

a *P < 0.01 vs. TIME 1*.

There was no effect (*P* = 0.68) of the different types of student-donkey interactions on student CCAS scores as shown in Table 2.

**Table 2:** Combined student CCAS scores during different types of student-donkey interactions.

	***n***	**Hands-off learning**	**Hands-on passive learning**	**Hands-on active learning**
CCAS scores	9	28.9 ± 13.5	32.4 ± 9.1	30.4 ± 14.7

*Student-donkey interactions included 30 min sessions during times 1 (week 2), 2 (weeks 5–8), and 3 (week 12) over three, 15-week periods*.

### Donkey Physiological Outcome

There was an effect (*P* = 0.02) of age on post treatment saliva cortisol concentrations. Mean concentrations were higher in immature (3.6 ± 4.9 nmole/L) compared to adult (1.7 ± 3.6 nmole/L) donkeys. Due, in part, to fewer number of adult donkeys (*n* = 5) and lack of complete data of pre- and post-sampling across the different types of interactions, only immature donkeys (*n* = 9) with complete data of pre- and post-sampling by 0900 h were evaluated in subsequent analyses.

Regardless of the type of student-donkey interaction, there was no effect (*P* = 0.06) on mean concentrations of saliva cortisol pre- vs. post-sampling as shown in Table 3. However, within the pre- and post-samplings, there was an effect (*P* = 0.02) of the type of student-donkey interaction on cortisol. Concentrations were lower (*P* < 0.05) in the hands-on passive and active learning interactions compared to the hands-off learning interactions.

**Table 3:** Combined immature donkey saliva concentrations of cortisol pre- and post-sampling in association with different student-donkey interactions during times 1 (week 2), 2 (weeks 5–8), and 3 (week 12) over three, 15-week periods.

**Cortisol (nmol/L)**	***n***	**Hands-off learning**	**Hands-on passive learning**	**Hands-on active learning**
Pre-sampling	9	2.1 ± 0.9	1.4 ± 0.4^a^	1.5 ± 0.8^a^
Post-sampling	9	3.8 ± 3.2	1.5 ± 0.6^b^	1.9 ± 0.6^b^

a *P < 0.05 vs. Pre-treatment of hands-off learning*.

b *P < 0.05 vs. Post-treatment of hands-off learning*.

## Discussion

The novelty of the human-animal interaction results observed herein provide preliminary information that necessitates further investigation surrounding the mutual benefits of wellbeing in veterinary school students and donkeys involved in student-donkey interactions.

There was no apparent change in student-donkey bonding regardless of the different levels of student-donkey interactions over time. The CCAS results correlated with informal qualitative behavioral assessments reported by the students which often included animal behaviors such as biting and kicking as well as donkeys being described as agitated, stubborn, and uncooperative. A student described an interaction as follows: “the donkey seemed like he was hungry or angry and literally felt being harassed for treats. The donkey bit my belly because he knows where I put the treats as he was trying to get them from my pocket. I stepped out after this.” Student descriptions and interpretation of their donkey interactions often demonstrated lack of knowledge surrounding normal donkey behavior and the importance of including such instruction within the curriculum. Furthermore, evidence validates the use of qualitative assessments in interpreting behavioral testing as well as links novice and potentially nervous handlers with equally fearful and nervous animals. Such can potentially explain the apparent stressful interactions and responses for both students and donkeys and implications that must be considered when participating in human-animal interactions and the importance of incorporating qualitative assessments in future student-donkey studies (50, 51). Additionally, although veterinary students reported on how important the concept of the human-animal bonding had in their decision of becoming a veterinarian, such changes can actually decrease during veterinary school experience, which parallels our observations while emphasizing the complexity of assessing such an interaction (52). Unfortunately, we are also limited as to the students' prior experience and comfort level with donkeys and large animals in general; factors which could have also contributed to the results. Further studies will require students having a baseline knowledge of donkey behavior prior to study.

Regardless of whether first-year students were paired with a donkey or not, their mean PSWQ scores decreased over time within a semester. Although not statistically different over time within either the interaction or control group, the overall main effect of time indicated student worry or stress was lower during Week 12 compared to Week 2. Generally, these results would be expected over time as first-year students in both groups would have experienced particularly high levels of stress upon entry to veterinary school which may be associated with homesickness, new social interactions, as well as lack of effective time management and study skills as they transition to the rigorous expectations of veterinary programs. Future studies are required to determine if students with or without animals in their lives with minimal interactions are as effective in reducing worry compared to students who have extensive interactions with animals. It would also be important to establish a baseline level of student stress levels prior to assessing the effects of student-donkey interactions.

While no attempt was made to evaluate student saliva cortisol concentrations at this time, there were no significant differences in immature donkeys between pre- and post-concentrations within any of the three types of student-donkey interactions within semester. However, there was a significant effect of the type of interaction. Mean concentrations were higher in association with the hands-off learning interaction compared to the hands-on passive and active interactions. Higher cortisol concentrations during the beginning of the semester and lower concentrations during the end of the semester associated with student physical interactions appears aligned with numerous studies (38–41) between dogs and their owners where cortisol concentrations deceased in association with non-noxious interactions (e.g., talking and touching). Future studies are required to clarify if the decrease in cortisol was a result of human physical contact, adaptive response over time to the environment or human presence, or a combination of both. Correspondingly, as previously indicated, behavioral assessment that details the donkeys' responsiveness, alertness, interest, avoidance or aggression can add important insight to endocrinological or other physiological (e.g., blood pressure, respiration) changes and could indicate a strong predictor of the wellbeing of the animal (21, 53).

In conclusion, a decrease in concentrations of saliva cortisol in immature donkeys during student-donkey interactions involving hands-on activities occurring later during the 15-week semester calls for more comprehensive studies with greater sample sizes which follow a randomized-control method for both human participants and animal subjects to truly clarify and quantify the apparent mutual benefits of wellbeing in veterinary school students and donkeys. Furthermore, extending the use of donkeys within veterinary school settings beyond the role of teaching animals but as a potential therapeutic source offers an opportunity in identifying the advantages that donkeys possess over other species in enhancing the wellbeing of veterinary students. Within this therapeutic capacity it is equally essential that veterinary universities further invest in educating their students on donkey behavior and continue to improve large animal welfare.

## Data Availability Statement

The raw data supporting the conclusions of this article will be made available by the authors, without undue reservation.

## Ethics Statement

The studies involving human participants were reviewed and approved by Ross University School of Veterinary Medicine Institutional Review Board. The students/participants provided their written informed consent to participate in this study. The animal study was reviewed and approved by Ross University School of Veterinary Medicine Institutional Animal Care and Use Committee.

## Author Contributions

EA developed the study design, oversaw data collection, supported analysis and interpretation, and lead the manuscript preparation. PH contributed to the design of the human–animal interactions and manuscript preparation. MM ran all donkey-saliva analysis and contributed to the manuscript preparation. DE was responsible for the statistical analyses and contributed to the manuscript preparation. JB was responsible for student-data interpretation and contributed to the manuscript preparation. MP contributed to data collection and manuscript preparation. JC oversaw the field activities and contributed to the manuscript preparation. DB contributed to the study design, responsible for the donkey-saliva data and interpretation, and contributed to manuscript preparation. All authors are accountable for the content of the work.

## Conflict of Interest

The authors declare that the research was conducted in the absence of any commercial or financial relationships that could be construed as a potential conflict of interest.

## Publisher's Note

All claims expressed in this article are solely those of the authors and do not necessarily represent those of their affiliated organizations, or those of the publisher, the editors and the reviewers. Any product that may be evaluated in this article, or claim that may be made by its manufacturer, is not guaranteed or endorsed by the publisher.

## References

[1] Hafen M Ratcliffe GC Rush BR. Veterinary medical student well-being: depression, stress, and personal relationships. J Vet Med Educ. (2013) 40:296–302. 10.3138/jvme.1112-101R 23975073

[2] McArthur M Mansfield C Matthew S Zaki S Brand C Andrews J. Resilience in veterinary students and the predictive role of mindfulness and self-compassion. J Vet Med Educ. (2017) 44:106–15. 10.3138/jvme.0116-027R1 28206835

[3] McArthur ML Andrews JR Brand C Hazel SJ. The prevalence of compassion fatigue among veterinary students in australia and the associated psychological factors. J Vet Med Educ. (2017) 44:9–21. 10.3138/jvme.0116-016R3 28206848

[4] Karaffa KM Hancock TS. Mental health experiences and service use among veterinary medical students. J Vet Med Educ. (2019) 46:449–58. 10.3138/jvme.1017-145r1 30806561

[5] Weston JF Gardner D Yeung P. Stressors and protective factors among veterinary students in New Zealand. J Vet Med Educ. (2017) 44:22–8. 10.3138/jvme.0116-014R1 28206841

[6] Cardwell JM Lewis EG. Vocation, belongingness, and balance: a qualitative study of veterinary student well-being. J Vet Med Educ. (2017) 44:29–37. 10.3138/jvme.0316-055R 28206847

[7] Cake MA Bell MA Bickley N Bartram DJ. The life of meaning: a model of the positive contributions to well-being from veterinary work. J Vet Med Educ. (2015) 42:184–93. 10.3138/jvme.1014-097R1 26075621

[8] Timmins RP. The contribution of animals to human well-being: a veterinary family practice perspective. J Vet Med Educ. (2008) 35:540–4. 10.3138/jvme.35.4.540 19228906

[9] Wollrab T. Human-animal bond issues. J Am Vet Med Assoc. (1998) 212:1675.

[10] Beetz A Uvnäs-Moberg K Julius H Kotrschal K. Psychosocial and psychophysiological effects of human-animal interactions: the possible role of oxytocin. Front Psychol. (2012) 3:234. 10.3389/fpsyg.2012.00234 22866043 PMC3408111

[11] Kamioka H Okada S Tsutani K Park H Okuizumi H Handa S. Effectiveness of animal-assisted therapy: a systematic review of randomized controlled trials. Complement Ther Med. (2014) 22:371–90. 10.1016/j.ctim.2013.12.016 24731910

[12] McCune S Kruger KA Griffin JA Esposito L Freund LS Hurley KJ. Evolution of research into the mutual benefits of human–animal interaction. Anim Front. (2014) 4:49–58. 10.2527/af.2014-0022

[13] Holmes C Goodwin D Redhead E Goymour K. The benefits of equine-assisted activities: an exploratory study. Child Adolesc Soc Work J. (2012) 29:111–22. 10.1007/s10560-011-0251-z

[14] Kern JK Fletcher CL Garver CR Mehta JA Grannemann BD Knox KR. Prospective trial of equine-assisted activities in autism spectrum disorders. Altern Ther Health Med. (2011) 17:14–20. 22164808

[15] Klontz B Bivens A Leinart D Klontz T. The effectiveness of equine-assisted experiential therapy: results of an open clinical trial. Soc Anim. (2007) 15:257–67. 10.1163/156853007X217195

[16] Frewin K Gardiner B. New age or old sage? A review of equine assisted psychotherapy. Aust J Counsel Psychol. (2005) 6:13–7.

[17] O'Haire ME. Animal-assisted intervention for autism spectrum disorder: a systematic literature review. J Autism Dev Disord. (2013) 43:1606–22. 10.1007/s10803-012-1707-5 23124442

[18] Rhett RB Grandjean PW. The efficacy of equine-assisted activities and therapies on improving physical function. J Altern Complement Med. (2016) 22:9–24. 10.1089/acm.2015.0171 26654868

[19] De Rose P Cannas E Reinger Cantiello P. Donkey-assisted rehabilitation program for children: a pilot study. Ann Ist Super Sanita. (2011) 47:391–6. 10.4415/ANN_11_04_11 22194074

[20] Panzera M Alberghina D Statelli A. Ethological and physiological parameters assessment in donkeys used in animal assisted interventions. Animals. (2020) 10:1867. 10.3390/ani10101867 33066258 PMC7602119

[21] Adamle KN Riley TA Carlson T. Evaluating college student interest in pet therapy. J Am Coll Health. (2009) 57:545–8. 10.3200/JACH.57.5.545-548 19254896

[22] Young JS. Pet therapy: dogs de-stress students. J Christ Nurs. (2012) 29:217–21. 10.1097/CNJ.0b013e31826701a7 23082615

[23] Somervill JW Kruglikova YA Robertson RL Hanson LM MacLin OH. Physiological responses by college students to a dog and a cat: implications for pet therapy. North Am J Psychol. (2008) 10:519–28. 10.1097/00005053-198710000-00005 3655768

[24] Blakeway S. The multi-dimentional donkey in landscapes of donkey-human interaction. Beyond Anthropocentr. (2014) 2:59–70. 10.7358/rela-2014-001-blak

[25] Burden F Thiemann A. Donkeys are different. J Equine Vet Sci. (2015) 35:376–82. 10.1016/j.jevs.2015.03.005

[26] Yilmaz O Wilson RT. The domestic livestock resources of Turkey: notes on donkeys. J Anim Plant Sci. (2013) 23:651–6. 10.1007/s11250-011-9957-3 21870064

[27] Dalla Costa E Dai F Murray LAM Guazzetti S Canali E Minero M. A study on validity and reliability of on-farm tests to measure human–animal relationship in horses and donkeys. Appl Anim Behav Sci. (2015) 163:110–21. 10.1016/j.applanim.2014.12.007

[28] Grandin T. Assessment of stress during handling and transport2. J Anim Sci. (1997) 75:249–57. 10.2527/1997.751249x 9027573

[29] Schmidt A Möstl E Wehnert C Aurich J Müller J Aurich C. Cortisol release and heart rate variability in horses during road transport. Horm Behav. (2010) 57:209–15. 10.1016/j.yhbeh.2009.11.003 19944105

[30] Palmer R Custance D. A counterbalanced version of Ainsworth's Strange Situation Procedure reveals secure-base effects in dog– human relationships. Appl Anim Behav Sci. (2008) 109:306–19. 10.1016/j.applanim.2007.04.002

[31] Möstl E Palme R. Hormones as indicators of stress. Domest Anim Endocrinol. (2002) 23:67–74. 10.1016/S0739-7240(02)00146-7 12142227

[32] Pendry P Smith AN Roeter SM. Randomized trial examines effects of equine facilitated learning on adolescents' basal cortisol levels. Hum Anim Interact Bull. (2014) 2:80–95.

[33] Prato-Previde E Custance DM Spiezio C Sabatini F. Is the dog-human relationship an attachment bond? An observational study using Ainsworth&apos;s strange situation. Behaviour. (2003) 140:225–54. 10.1163/156853903321671514

[34] Topál J Miklósi Á Csányi V Dóka A. Attachment behavior in dogs (Canis familiaris): a new application of Ainsworth's (1969) strange situation test. Int J Comp Psychol. (1998) 112:219–29. 10.1037/0735-7036.112.3.219 9770312

[35] Koolhaas JM de Boer SF Coppens CM Buwalda B. Neuroendocrinology of coping styles: towards understanding the biology of individual variation. Front Neuroendocrinol. (2010) 31:307–21. 10.1016/j.yfrne.2010.04.001 20382177

[36] Ranabir S Reetu K. Stress and hormones. Indian J Endocrinol Metab. (2011) 15:18–22. 10.4103/2230-8210.77573 21584161 PMC3079864

[37] Odendaal JSJ Meintjes RA. Neurophysiological Correlates of Affiliative Behaviour between Humans and Dogs. Vet J. (2003) 165:296–301. 10.1016/S1090-0233(02)00237-X 12672376

[38] Miller SC Kennedy CC DeVoe DC Hickey M Nelson T Kogan L. An examination of changes in oxytocin levels in men and women before and after interaction with a bonded dog. Anthrozoös. (2009) 22:31–42. 10.2752/175303708X390455

[39] Handlin L Hydbring-Sandberg E Nilsson A Ejdebäck M Jansson A Uvnäs-Moberg K. Short-term interaction between dogs and their owners: effects on oxytocin, cortisol, insulin and heart rate—an exploratory study. Anthrozoös. (2011) 24:301–15. 10.2752/175303711X13045914865385

[40] Handlin L Nilsson A Ejdebäck M Hydbring-Sandberg E Uvnäs-Moberg K. Associations between the psychological characteristics of the human–dog relationship and oxytocin and cortisol levels. Anthrozoös. (2012) 25:215–28. 10.2752/175303712X13316289505468

[41] Petersson M Uvnäs-Moberg K Nilsson A Gustafson L-L Hydbring-Sandberg E Handlin L. Oxytocin and cortisol levels in dog owners and their dogs are associated with behavioral patterns: an exploratory study. Front Psychol. (2017) 8:1796. 10.3389/fpsyg.2017.01796 29081760 PMC5645535

[42] Meyer TJ Miller ML Metzger RL Borkovec TD. Development and validation of the Penn State worry questionnaire. Behav Res Ther. (1990) 28:487–95. 10.1016/0005-7967(90)90135-6 2076086

[43] Molina S Borkovec TD. The Penn State Worry Questionnaire: psychometric properties and associated characteristics. In: Davey GCL Tallis F, editors. Worrying: Perspectives on Theory, Assessment and Treatment. John Wiley & Sons (1994). p. 265–83.

[44] Zasloff RL. Measuring attachment to companion animals: a dog is not a cat is not a bird. Appl Anim Behav Sci. (1996) 47:43–8. 10.1016/0168-1591(95)01009-2

[45] Castelli P Hart LA Zasloff RL. Companion cats and the social support systems of men with AIDS. Psychol Rep. (2001) 89:177–87. 10.2466/pr0.2001.89.1.177 11729540

[46] Fell LR Shutt DA Bentley CJ. Development of a salivary cortisol method for detecting changes in plasma “free” cortisol arising from acute stress in sheep. Aust Vet J. (1985) 62:403–6. 10.1111/j.1751-0813.1985.tb14120.x 3833194

[47] Cooper TR Trunkfield HR Zanella AJ Booth WD. An enzyme-linked immunosorbent assay for cortisol in the saliva of man and domestic farm animals. J Endocrinol. (1989) 123:R13–6. 10.1677/joe.0.123R013 2607243

[48] Bonelli F Rota A Aurich C Ille N Camillo F Panzani D. Determination of salivary cortisol in donkey stallions. J Equine Vet Sci. (2019) 77:68–71. 10.1016/j.jevs.2019.02.027 31133319

[49] Broman KW Woo KH. Data organization in spreadsheets. Am Stat. (2018) 72:2–10. 10.1080/00031305.2017.1375989

[50] Minero M Tosi MV Canali E Wemelsfelder F. Quantitative and qualitative assessment of the response of foals to the presence of an unfamiliar human. Appl Anim Behav Sci. (2009) 116:74–81. 10.1016/j.applanim.2008.07.001

[51] Ellingsen K Coleman GJ Lund V Mejdell CM. Using qualitative behaviour assessment to explore the link between stockperson behaviour and dairy calf behaviour. Appl Anim Behav Sci. (2014) 153:10–7. 10.1016/j.applanim.2014.01.011

[52] Martin F Ruby K Farnum J. Importance of the human-animal bond for pre-veterinary, first-year, and fourth-year veterinary students in relation to their career choice. J Vet Med Educ. (2003) 30:67–72. 10.3138/jvme.30.1.67 12733098

[53] Gonzalez-De Cara CA Perez-Ecija A Aguilera-Aguilera R Rodero-Serrano E Mendoza FJ. Temperament test for donkeys to be used in assisted therapy. Appl Anim Behav Sci. (2017) 186:64–71. 10.1016/j.applanim.2016.11.006

